# Supplementary material for: The toxicity assessment of phosmet on development, reproduction, and gene expression in Daphnia magna
Source: PeerJ. 2024 Feb 28;12:e17034. doi: 10.7717/peerj.17034 (PMC10908259; doi:10.7717/peerj.17034)
Supplement: Supplemental Information 2 [file peerj-12-17034-s002.docx]

**The toxicity assessment of phosmet on development, reproduction, and gene expression in *Daphnia magna***

Mustafa Atas^1^, Ceyhun Bereketoglu^2,3*^

1. Iskenderun Technical University, Institute of Graduate Studies, Managing Chemical, Biological, Radioactive, Nuclear Risks, Hatay, Turkey
2. Iskenderun Technical University, Faculty of Engineering and Natural Sciences, Department of Biomedical Engineering, Hatay, Turkey
3. Department of Bioengineering, Faculty of Engineering, Marmara University, 34722, Istanbul, Turkey.

*Corresponding author: Ceyhun Bereketoglu, Email: ceyhun.bereketoglu@marmara.edu.tr


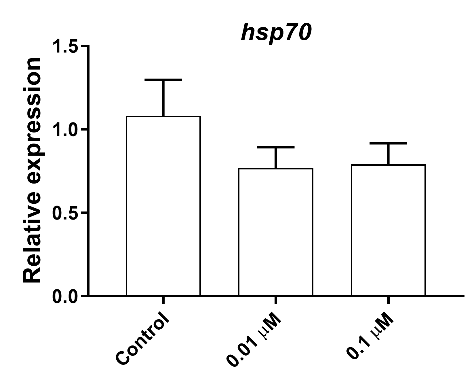

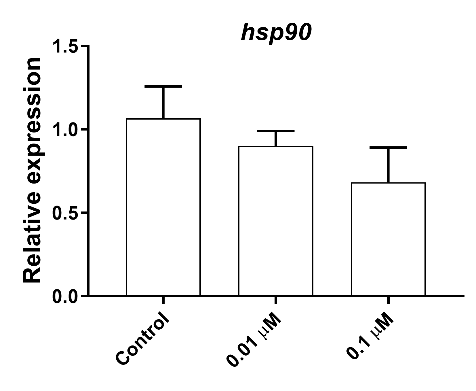

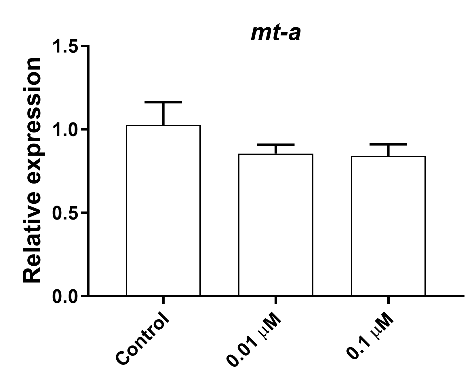

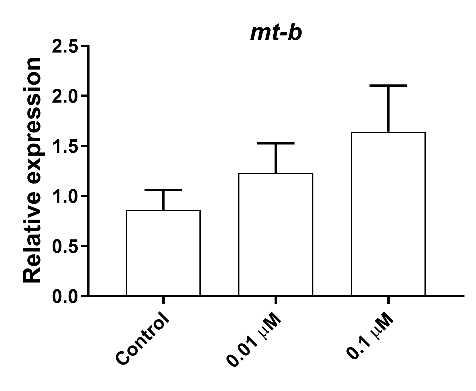

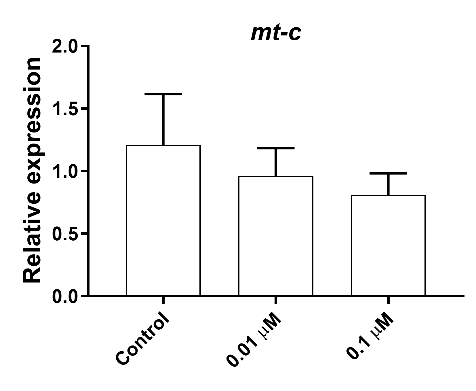

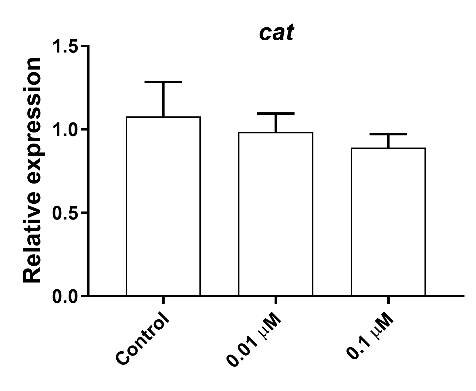

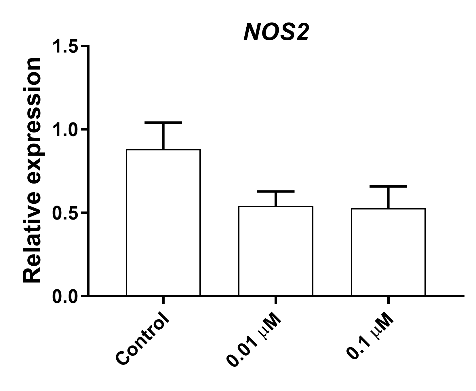

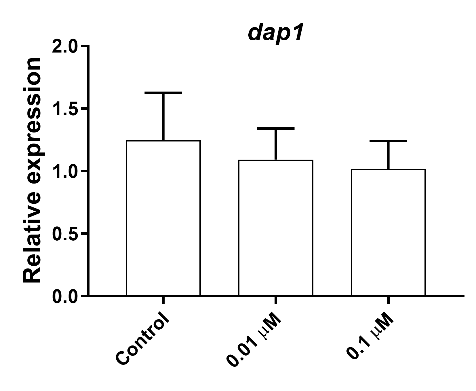

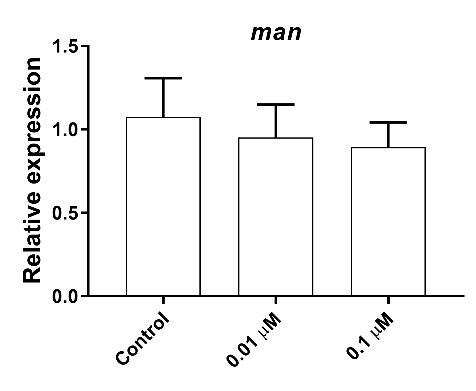

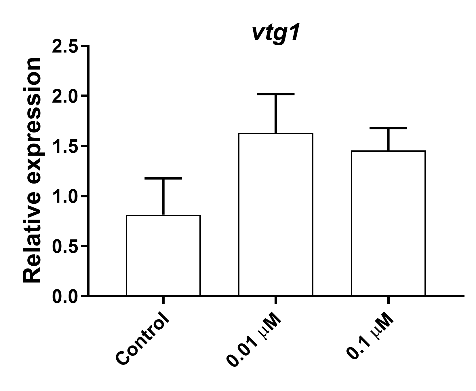


**Fig.S1. The genes did not show significant expression changes*.*** *D. magna* neonates (<24 h old) were exposed to 0.01 and 0.1μM phosmet for 24 h, and the gene expression levels were determined using qPCR. Statistical analyses were performed using one-way ANOVA followed by Dunnett post-test and the difference were accepted significant if p values < 0.05. n = 5.


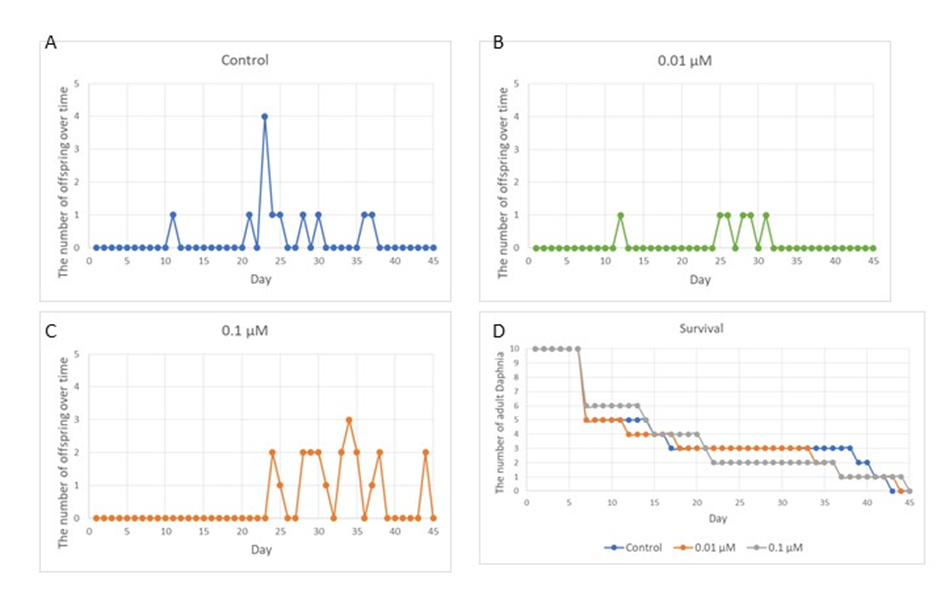


**Fig.S2. The brood number in the different treatments over time.** Daphnia magna neonates (˂ 24 h old) were exposed to phosmet (0.01 and 0.1 µM) and number of offspring was recorded daily until all organisms were dead. For each concentration, 10 organisms were used and experiments were performed in triplicates. The number of offspring and dead organisms were recorded daily and removed. (A) The control, (B) 0.01 µM, (C) 0.1 µM, and (D) Survival.
